# Dietitian reflections on video consultations: a descriptive qualitative study

**DOI:** 10.1186/s12913-026-14818-2

**Published:** 2026-05-28

**Authors:** Sarah Persson, Karin Danielsson, Petra Rydén, Anette Edin-Liljegren, Cecilia Olsson

**Affiliations:** 1 Department of Food, Nutrition and Culinary Science, Umeå University, Universitetstorget 4, Umeå, 901 87 Sweden; 2 The Swedish Centre for Rural Health, Region Västerbotten, Stationsgatan 3, Storuman, 923 31 Sweden; 3 Department of Informatics, Umeå University, Universitetstorget 4, Umeå, 901 87 Sweden; 4 Department of Epidemiology and Global Health, Umeå University, Universitetstorget 4, Umeå, 901 87 Sweden

**Keywords:** Health personnel, Health services accessibility, Qualitative research, Remote consultation, Telehealth

## Abstract

**Background:**

A transition is seen in healthcare from routine face-to-face consultations to increased reliance on video consultations. Practice by healthcare professionals, who serve extensive geographical areas and conduct relatively few physical examinations, may particularly benefit from this modality. While video consultations attract political interest by offering practical advantages—such as reduced travel—healthcare professionals remain responsible for maintaining care quality. Thus, it is essential to examine how healthcare professionals, such as registered dietitians, reason about the use of video consultations to better support informed decisions about the use of video consultations in healthcare practice.

**Methods:**

Data were collected using digital interviews from nine registered dietitians selected using purposeful, convenience, and snowball sampling. The data were analysed using reflexive thematic analysis, guided by a patient-centred framework for access to healthcare that considers both patient and provider perspectives.

**Results:**

Dietitians reported that video consultations enhanced flexibility and access to healthcare, though not universally for all patients or stages of care. Three themes emerged: *Contributing to good and accessible healthcare*; *Gaining control over one’s working situation*; and *Keeping the care process in mind*. Dietitians selected video consultations based on considerations of equity and effectiveness but preferred face-to-face consultations when physical assessments were necessary, or when patients lacked adequate digital skills. This was interpreted as personal preferences and normative responses to organizational structures, and policy frameworks.

**Conclusion:**

When multiple aspects of care delivery are considered simultaneously, perceived benefits may overshadow limitations, and vice versa. Future research should explore how to support healthcare professionals in involving patients and navigating competing policy frameworks, professional responsibilities, and contextual demands when deciding on consultation modalities.

**Supplementary Information:**

The online version contains supplementary material available at 10.1186/s12913-026-14818-2.

## Key findings

Healthcare professionals justified their choice of video consultation based on the perception of providing equal care, and the belief that it enhanced patients’ access to care.

A need for anthropometrical measurements, and patient’s lack of digital literacy were argued as reasons for choosing other consultation modalities than video.

Transitioning to video consultations without addressing person-centeredness and treatment quality might lead to lapses in health outcomes.

**Supplementary Information:**

The online version contains supplementary material available at 10.1186/s12913-026-14818-2.

## Background

A large part of the world, including many sectors of society such as healthcare, faces a shift from routine face-to-face consultations (FTFCs) towards a bigger reliance on information communication technology alternatives such as video consultations (VCs) [1] and telephone consultations (TCs), collectively referred to as telehealth when used within healthcare [2]. While FTFCs and TCs have been around for many years, VCs are quite new. VC refers to a synchronous video-mediated consultation between a patient and a healthcare professional [1–3]. The World Health Organisation (WHO) and healthcare organisations worldwide are promoting telehealth to increase access to healthcare because of its flexibility of geographical location and also for the possibility to reduced inequalities [4–9]. Moreover, by minimizing travel requirements, telehealth could also provide environmental advantages and reduce costs [10–12]. However, challenges in forming therapeutic relationships with patients and managing assessments such as physical examinations and blood tests are raised as concerns about the suitability of telehealth, often restricting the use to follow-up consultations [13–15]. Additionally, challenges associated with VCs include accessibility for various groups, levels of digital literacy, and availability of adequate technical equipment [6, 16–18]. Thus, the suitability of VC is not uniform across healthcare professions practices. It depends on both the clinical requirements and the technological infrastructure necessary to ensure delivery of good quality care. Professions where the care process is predominantly conversational, such as dietetics and psychology, are particularly well aligned with the functional characteristics of VC [19, 20]. These interactions rely less on physical examinations and more on verbal engagement, making them inherently compatible with remote modalities [19, 20]. Considering this, Registered Dietitians (RDs) were selected in the present study as a representative profession of healthcare professionals to explore the implementation and perceived appropriateness of video consultations within routine clinical practice.

People‑Centred Health Care (PCHC), Person‑Centred Care (PCC), and Patient‑Centred Care (PaCC) aim to prioritize individual needs, improve health outcomes, and enhance patient satisfaction [21–23], emphasizing holistic care, active participation, empowerment, and respectful communication. PCHC, endorsed by the WHO, extends beyond clinical settings to include individuals, families, communities, and healthcare professionals [22]. PCC builds on PaCC by supporting the whole person and promoting a meaningful life beyond clinical outcomes [23]. In Sweden, PCC is promoted within the ongoing national healthcare reform ‘Good quality and local health care’, aiming at achieving good‑quality and equal healthcare through, among other measures, increased accessibility [24], and is increasingly implemented in regional healthcare practice [25]. While PCC can be applied in virtual care [13], further research is needed to understand how PCC principles are perceived and implemented in digital settings.

Levesque and colleagues describe patient-centred access to healthcare as encompassing five dimensions [26], here seen through the lens of VC use: A*pproachability* and *ability to perceive* healthcare relates to how visible and understandable healthcare services are for the patient, shaping whether patients are presented with the modality of VC when needing care. *Acceptability* and *ability to seek* healthcare involves how well services align with patients’ and RDs’ cultural and social expectations, influencing their willingness to *seek* and to offer VCs. *Availability and accommodation* together with *ability to reach* healthcare concern the prerequisites for care, which determine whether VC is a possible option as meeting modality. *Affordability* and *ability to pay* for healthcare refers to whether individuals can afford the cost of healthcare by VCs, which directly affects their ability to use it. Lastly, *appropriateness* and *ability to engage* with healthcare is about how well services such as VCs meet the specific needs of patients, impacting their ability to engage with healthcare. VCs may help improve access to healthcare across all these dimensions, although more research is needed to fully understand how they influence each aspect.

The availability of dietetic services in Sweden is limited because of the low ratio of RDs, difficulties in hiring, and the concentration of services in centralized locations [27]. Consequently, many rural areas do not have access to a local dietetic service. This study is part of the Dietitian Online (DiOn) research project [28], which seeks to enhance understanding of, and assess the impact of VCs, in Swedish healthcare from the viewpoints of patients, RDs and the broader community. The Nutrition Care Process model with Nutrition Assessment and Reassessment, Nutrition Diagnosis, Nutrition Intervention, and Nutrition Monitoring and Evaluation is designed to help RDs in their work process to achieve mutual standards, to increase clarity in clinical practices, and to have a PCHC approach [29]. VCs have gained political traction due to their practical benefits, such as reduced travel and increased accessibility [4, 5, 24, 30]. For RDs, VCs offer opportunities to enhance nutritional care through improved continuity and reach [31–34], which could enable earlier treatment and better treatment outcomes. Work life benefits such as increased flexibility and added ease of scheduling patient visits are also reported [35–37]. Also, as dietetic practices largely focus on communication to promote behaviour change, VC has the potential to be equally effective as FTFCs [38]. However, VCs pose challenges to traditional practices for RDs such as anthropometric measurements and requirements for technical equipment and ability [35, 39–41]. Despite these challenges, the COVID-19 pandemic demonstrated the value of VCs in maintaining continuity of care, enabling treatments that might otherwise have been postponed or limited to TC only [35, 42]. The experience and knowledge of using VCs as a substitute can enable VCs as an integrated alternative as meeting modality in healthcare practice, but successful large-scale implementation requires an understanding of healthcare professionals’ experiences. To support informed decisions about when and how to use VCs in dietetic practice, it is important to understand how RDs approach the use of VCs. The aim of this study was to increase understanding of how RDs reasoned about the use of VCs during a one-year period that partially overlapped with social distancing recommendations related to the COVID‑19 pandemic restrictions, and how these reflections were shaped by organizational structures, policy frameworks, professional normativity, and individual drivers.

## Methods

### Study design

A descriptive qualitative design was adopted to produce a straightforward and comprehensive account of the phenomenon of interest grounded in participants’ accounts [43, 44]. The Qualitative Longitudinal Research (QLR) approach was chosen to capture time-dependent changes during the COVID-19 pandemic including shifts in social distancing recommendations based on authorities assessments of the epidemiological situation with anticipated effect on clinical practice and the choice of consultation modality [45, 46]. The reporting of findings from this study follows the Consolidated Criteria for Reporting Qualitative Research (COREQ) [47].

### Research setting and participants

The study was situated within the Swedish health care system, publicly funded. Dietetic care in Sweden is commonly delivered through the public healthcare system, either in primary care (outpatient clinics) or hospitals (both inpatient and outpatient), with only a small number of dietitians working in private practice. Participants for the interviews were recruited through voluntary sampling [48], which included a mix of purposeful sampling, convenience sampling, and snowball sampling [49]. To be eligible for the study, participants needed to be clinically active as a dietitian in Sweden and to have experience of using VCs. Exclusion criteria included RDs without clinical experience of video consultations, those working exclusively in non-clinical roles, and individuals unable to participate in a Swedish or English-language interview, or who withdrew consent. Participants were recruited in the spring of 2021 through an advertisement in the Swedish Association of Clinical Dietitians’ e-mail newsletter, and via selected Facebook groups directed to RDs in Sweden. The advertisement included information regarding the aim and outline of the study with two interview occasions, as well as information about the responsible researchers. Twenty-one RDs volunteered and were interviewed. Twelve RDs dropped out before the second interview occasion. The participants withdrew for reasons such as changes in position, parental leave, or management directives. In line with QLR, their initial interviews were excluded from the analysis, leaving nine participating RDs who participated in both interviews. Sample size was guided by information power rather than predetermined numerical targets [50]. The longitudinal interview design, the specificity of the sample, and the depth of the interviews were considered to provide sufficient information to address the study aim.

### Data collection and management

Initial background data were collected when respondents registered for participation using the survey tool Survey & Report from Artologik^®^. Collected data used in this study were: frequency of video modality use; attitude regarding VCs in clinical practice; years of RD-work experience; working field (in- or outpatient care); and healthcare region.

This study is based on semi-structured interviews with RDs, all but one working in public healthcare in Sweden. Each RD was interviewed twice. The first interview was conducted in the spring of 2021 and the second in spring of 2022. The second interview also served as a complementary occasion for additional in-depth inquiries related to empirical data from the 2021 interviews, and to monitor developments in the field.

In 2021, a semi-structured interview guide (Interview guide 2021) was constructed covering different areas related to dietetic practice in relation to telehealth in general and VC in particular. The areas covered the impact of the pandemic on RDs’ clinical practice, and their use of VCs. The questions also explored RDs’ experiences with VCs, attitudes towards VCs, the interpersonal relationships during VCs, technical breakdowns, and their thoughts and wishes regarding future use of VCs. The interview guide was designed to facilitate open exploration of RDs’ experiences of video consultations without presupposing specific attitudes [51]. The interview guide was pilot tested with an RD within the research group prior to data collection, with one pilot interview, allowing refinement of questions and procedures [52]. Consequently, minor edits were made where questions with double inquiries were adjusted to single ones.

The semi-structured interview guide (Interview guide 2022) was revised for the 2022 interviews. Questions were asked regarding developments during the past year regarding clinical practice with VC usage, and attitudes regarding VC use. More in-depth questions were asked regarding perceptions of VC characteristics in relation to TCs and FTFCs, experiences of multiparticipant VCs, additional tools available for VCs such as screen sharing, and their thoughts for the future of practice.

Individual interviews were conducted using the VC video and audio recording tool provided by Umeå University. Most interviews were conducted jointly by SP and KD, with two exceptions where SP conducted the interviews alone. The use of two interviewers was intended to enhance reflexivity by bringing complementary perspectives into the interview process, allowing for complementary follow-up questions and interpretations to emerge [53]. Interviews conducted in 2021 ranged from 34 to 52 min in length (mean 43 min). In 2022, interview durations varied between 31 and 46 min (mean 38 min). The recorded interviews were then transcribed verbatim and anonymised. Field notes were taken by the interviewers throughout every interview session.

### Data analysis

#### Process of analysis

**Fig. 1:**
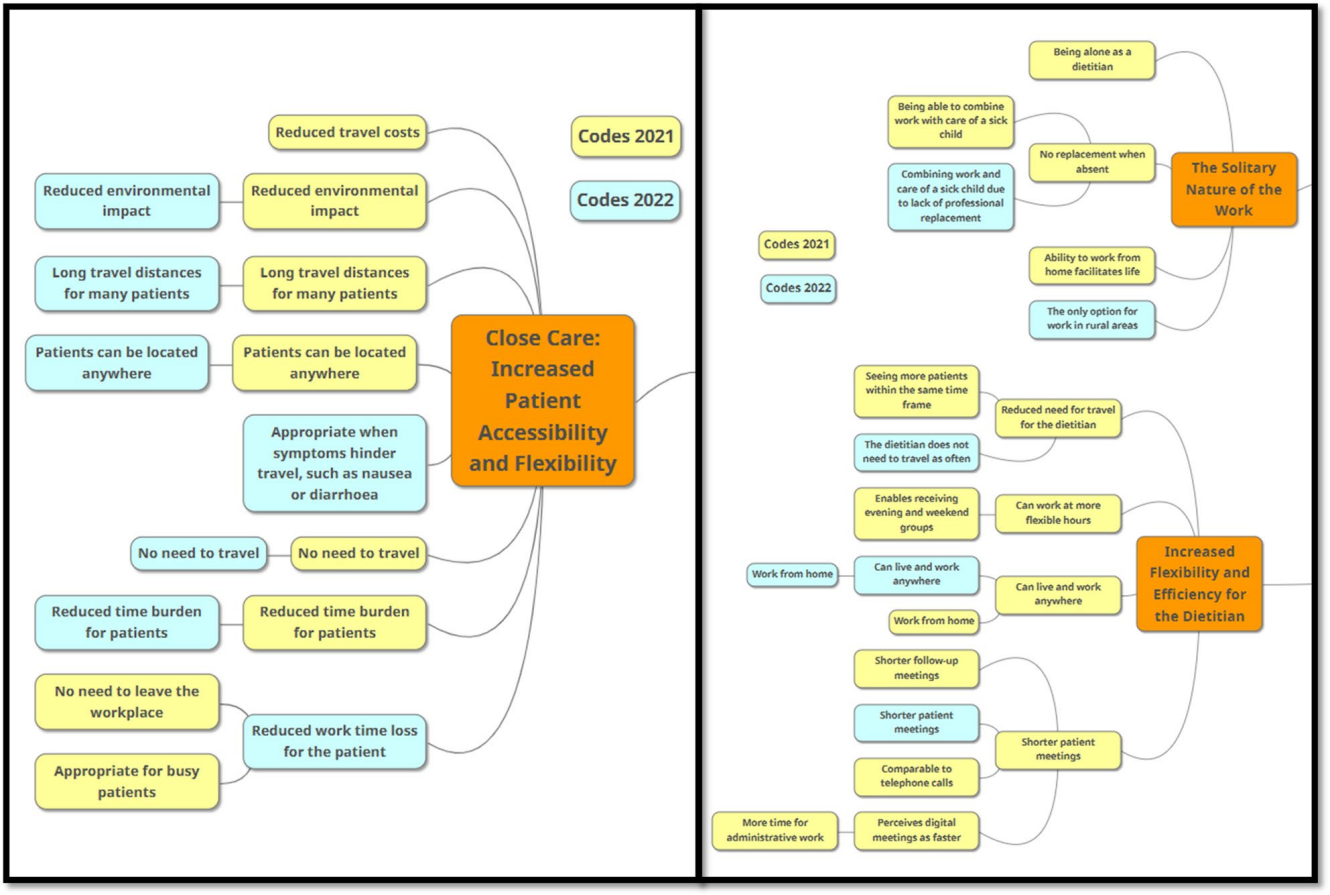
Examples illustrating the step in the analysis where codes were organized into clusters

Reflexive thematic analysis by Braun and Clarke (2022) was performed on those parts of the interview data that related to VC use by the RDs [53]. First SP thoroughly read and re-read all 9 transcribed interviews from 2021 and identified parts that dealt with how the RDs reasoned the use of VCs, as an area of interest for analysis. The analysis was conducted manually by SP in line with reflexive thematic analysis, with ©ATLAS.ti Scientific Software Development GmbH, used solely as a technical aid to support the organisation and coding of data [53]. All interviews from 2021 were inductively coded using an open coding technique, and clustered according to the study aim. Interviews from 2022 were subsequently coded in the same manner, and clustered together with the 2021 codes, from which preliminary themes were constructed. Code clustering across both data collections was conducted by SP using a mind-map format and served as a basis for iterative discussion with CO and KD (Fig. 1). Through repeated engagement with the coded data, clusters and candidate themes were examined, reworked and refined in a recursive process rather than developed in a linear manner.

In the next step of the analysis, SP revisited the codes to identify potential patterns of change between the 2021 and 2022 interviews, regarding reasoning about the VC choices, following the Qualitative Longitudinal Research (QLR) design [45, 46].

Single-author coding was conducted in line with reflexive thematic analysis and the notion of the subjective researcher, with reflexivity embedded through iterative memo-writing documenting analytical decisions and evolving interpretations. Coding was treated as a recursive and interpretive process. Co-working with additional researchers on clustering and thematization was chosen as collaborative sense-making not to reach consensus but to enrich the analysis [54]. The QLR [45, 46] was considered a way to follow a phenomenon over time, including potential changes in experiences and the context. The citations used were dated with the year of the interview.

Levesque et al.’s conceptual framework for patient-centred access to healthcare [26] was used to interpret the findings in relation to previous research. Four of the five dimensions of the framework were applied to interpret the implementation process of VCs. The fifth dimension, *Affordability* and *ability to pay*, was not included, as the empirical material did not provide sufficient data to meaningfully address this aspect of access.

### Trustworthiness and reflexivity

The Dietitian Online (DiOn) project by Umeå University and the Swedish Centre for Rural Health aimed to evaluate VCs’ effect on patients, RDs, and society [28, 55]. The research group includes expertise in food and nutrition, informatics, epidemiology, and rural health, as well as practical experience of dietetic clinical work and video consultations.

Trustworthiness was addressed through transparency, reflexive engagement, and contextualised analysis [56]. In line with reflexive thematic analysis, interpretations were developed through iterative engagement with the data, supported by memo‑writing and illustrative quotations. Reflexivity was maintained by ongoing attention to researcher positioning, and contextual descriptions enable assessment of transferability. Discussions with practising dietitians supported reflexive refinement rather than validation.

### Ethical considerations

The study was approved by the Swedish Ethical Review Authority (dnr 2015/330 − 31) and conducted in accordance with the Declaration of Helsinki. Ethical considerations included informed consent, voluntary participation, confidentiality, minimisation of potential harm, and secure handling and storage of data. Eligible participants responded to the study advertisement via an online form used to collect contact details and background information for purposive participant selection. When completing the form, participants were asked to indicate consent for the temporary storage of data for recruitment and selection purposes. Participants who completed the form and were eligible subsequently received a study information letter by email and were informed that replying to the email with confirmation of participation constituted written informed consent. Verbal consent was confirmed at the time of the interview.

## Results

### Participants

Nine RDs with public healthcare experience of VC use participated in this study. Participants were between 26 and 60 years old with most of them under 41 (*n* = 6). They had worked clinically as RDs from five to twenty years (mean 10 years) and worked in primary care settings (*n* = 6), mostly with outpatient care, or in hospital care settings (*n* = 3), with a combination of outpatient and inpatient care. Most RDs were women (*n* = 8). The RDs were employed in 7 out of the 21 healthcare regions in Sweden, spanning the north to the south of the country and including both urban and rural areas.

Four RDs held ambulatory positions with travel between primary care units, though travel was limited by pandemic restrictions. In 2021, four RDs were the sole RD at their unit, compared with two in 2022. Digital employment increased from two RDs in 2021 to three in 2022. The remaining RDs had on‑site employment, with varying possibility to working from home, one to two remote workdays per week was most common. All participants had opportunity to work from home to some extent, though none were required to do so; One RD chose not to work from home due to concerns about technical failures.

The prerequisites for, and attitudes about, VCs among the interviewed RDs are shown in Table 1. Most RDs reported that their debut with VC use occurred after the onset of the pandemic (*n* = 6) and all RDs expressed an overall positive attitude towards VCs. Moreover, some RDs (*n* = 2) had become increasingly positive about VCs by the 2022 interview, while one had become more sceptical because of doubts about the technical robustness of VCs in practice.

**Table 1:** Video consultation (VC) experiences and attitudes

Variable	Leo	Ruth	Dana	Emma	Julia	Annie	Rita	Alice	Elisabeth
Pre-pandemic VC debut	Yes	-	-	-	Yes	Yes	^1^	-	-
Positive VC attitude 2021	Yes	Yes	Yes	Yes	Yes	Yes	Yes	Yes	Yes
Change in VC attitude 2022^2^	→	↘	→	→	↗	↗	→	→	→
Possibility of screen sharing 2021	Yes	-	Yes^3^	-	-	-	-	Yes	Yes
Possibility of screen sharing 2022	Yes	-	Yes^3^	Yes^3^	Yes	-	Yes	Yes	Yes
Possibility of multi-party VC in 2021	Yes	-	Yes^3^	-	-	-	Yes^3^	Yes	Yes
Possibility of multi-party VC in 2022	Yes	-	Yes^3^	Yes^3^	Yes	-	Yes^3^	Yes	Yes
Frequency of VC use 2021	Daily	Daily	Daily	Daily	Weekly	Weekly	Weekly	Weekly	Monthly
Change in consultation modality from 2021 to 2022	Yes^4^	-	Yes^4^	Yes^4^	-	-	Yes^4^	Yes^4^	Yes^5^

^1^Unclear debut occasion

^2^→ = unchanged attitude, ↘ = decreased positivity, ↗ = increased positivity

^3^Not on platform for individual consultations, only for group consultations

^4^Increased face-to-face consultations

^5^Increased video consultations

In 2021, four RDs were able to use screen-sharing during VCs. In 2022, an additional three RDs reported having access to this function (Table 1). The possibility of having more than two parties in the VCs was reported by some RDs (*n* = 5) during the 2021 interviews, with a couple of additional RDs (*n* = 2) during the 2022 interviews.

The frequency of VC use varied among the RDs. Many of the RDs stated that they were following organizational directives to offer VCs to the patients if possible and had established protocols on how to do so. In 2021, four RDs reported using VCs every day, four every week, and one RD every month (Table 1). In the 2022 interviews, five RDs reported having more FTFCs than in 2021, replacing either TCs or VCs, while one RD reported increased VC use compared with 2021.

Three themes from RD interviews were constructed during the analysis: ‘Contributing to Good and Accessible Healthcare’, ‘Gaining Control Over One’s Working Situation’, and ‘Keeping the Care Process in Mind’ (Table 2). The first paragraph in each theme is a summary of the content. The following paragraphs represent clusters that build the foundation of the theme. Each cluster is based on codes from both the 2021 and 2022 interviews. At the end of each cluster paragraph, potentially observed differences between the two interview occasions are highlighted.

**Table 2:** Overview of clusters within the three overarching themes

Themes	Theme 1: Contributing to Good and Accessible Healthcare	Theme 2: Gaining Control Over One’s Working Situation	Theme 3: Keeping the Care Process in Mind
Clusters	- Close Care: Increased Patient Accessibility and Flexibility - Good Care: Care of Equal or Higher Quality - The Ambition and Challenge of Person‑Centred Care	- Concerns About a Reversal of Digitalisation - Greater Continuity in Dietetic Care - Improved Continuity from Patients - Increased Flexibility and Efficiency for the Dietitian - Individual Incentives for Digitalisation - Suitability of the Dietetic Profession - The Solitary Nature of the Work	- A Temporary Solution - Compensating Through Other Consultation Modalities - Digital Limitations - Patients’ Unfamiliarity with Digitalisation - Uncertainty Regarding Comprehension in Situations of Language Barriers - Uncertainty Regarding Patients’ Digital Literacy - Uncertainty Regarding Patients’ Expectations - Uncertainty Regarding Treatment Quality

### Theme 1: Contributing to good and accessible healthcare

The theme *Contributing to Good and Accessible Healthcare* highlights how the interviewed RDs described offering VCs to most patients as a first choice, with the aim of increasing flexibility and accessibility of nutritional care and, during the pandemic, also contributing to reductions in the possibility of disease transmission. This was interpreted as a personal preference combined with a genuine commitment to change and a normative professional positioning, where offering VCs was constructed as the correct and expected response, in line with national and international drivers for person‑centredness and sustainability. *Accessible* healthcare was recognized by independence from geographical distance, improving equity, and allowing care to be provided with more convenience for the patient. *Good* healthcare was recognized by offering equally good quality care in VCs as FTFCs but without the need to travel, and with shorter waiting times for the consultation. However, information about individual patient consultation modality preferences were recognized as challenging, and systematic evaluations of VC use were lacking.

VCs functioned as an enabler of increased flexibility for patients regarding the geographical location of treatment, thereby saving time and effort, and improving access to nutritional care. RDs described the long distances some patients were expected to travel to meet RDs and the relief patients expressed for not having to travel. This was even more relevant where patients had a physical status or symptoms that made it difficult or uncomfortable for them to travel to the healthcare unit. The economic benefits of reduced patient travel were also mentioned as a secondary positive effect of VCs compared to FTFCs, as were the economic benefits for healthcare units providing the service. Yet another positive effect of reduced travel was the time saved for patients, reducing the need to take time off work, or from their other responsibilities. Offering consultation times before and after office hours was also described as a future opportunity with VC use but was not yet offered at the time of the interviews. The belief in increased flexibility and access through VCs was equally present at both interview occasions, as illustrated here by Alice and Julia from the 2021 and the 2022 interview occasion respectively:

We have had patients who have also pointed this out, that they think it’s really nice to be allowed to take it [the consultation] digitally instead of having to set aside half a day to travel back and forth. Sometimes they have almost two hours of travel time one way to get here. So maybe the visit is 40 min and then they go back again, so it becomes a whole day. But now you don’t have to take as much time off from work and so on. (Alice, interview 2021)

Then they [the patients] don’t need to take so much time off from their job, for example, if they are attending an IBS school or a cooking school, or whatever it might be, they can just connect and participate. (Julia, interview 2022)

Good care was understood by RDs in various ways. For example, when they could offer patients appointments with shorter waiting times using VCs. This improvement was due to the ease of finding mutually acceptable times for consultation, not needing to be physically present, and allowing for more consultation appointments with less time spent traveling by ambulatory RDs. The associated risk of disease transmission during FTFCs because of the ongoing COVID-19 pandemic was also mentioned by the RDs, arguing that VCs were a safer alternative for the patients. Many of the RDs argued that VCs were well-suited for the type of counselling mainly used by Swedish RDs, involving conversations and with fewer physical examinations than RDs in other countries. One RD argued that, just as psychologists can use VCs for most counselling, so can RDs. The suitability of VCs was especially noted for follow-up appointments. When FTFCs had been conducted the VC follow-ups were perceived as more easily conducted as first measurements and assessments had already been made. Organized evaluations of patient experiences with VCs were conducted by one of the RD-clinics and was said to be yielding positive results. One noted difference in RD reasoning between the 2021 and the 2022 interviews was that the RDs reflected in more detail during the 2022 interview on how they perceived VCs as providing equally good healthcare as FTFCs. However, the argument for good quality was mainly based on the absence of patient complaints rather than from systematic quality assessments. Here described by Rita:

…before trying digital visits, many people probably feel uncertain about it or would prefer a regular visit if given the choice. But I think those who have had digital visits have been satisfied and feel that it is just as good as a physical visit. At least, that’s the feedback I’ve received. It’s rare for a patient who has had a digital visit to say, ‘No, I must come in person next time.’ Instead, it’s usually the opposite. (Rita, interview 2022)

For patients preferring not to meet the RD via VCs, it was still possible to have TCs or FTFCs instead. Although, longer waiting times were sometimes necessary for FTFCs in 2021, but less so in 2022 because restrictions had been lifted to some degree and more FTFCs were available. However, smaller differences were noted at RD units where VCs still were considered as first choice modality, here described by Leo:

I still think it’s pretty much at the same level, actually … I would say … because those [RDs] who can and want to use video, just like in previous years, have continued to do so now. And then for the consultations where physical visits are needed, or when they [the patients] have requested it, we have offered that. But that’s exactly what we did a year ago as well. I would say it’s pretty much the same. (Leo, interview 2022)

### Theme 2: Gaining control over one’s working situation

The theme *Gaining Control Over One’s Working Situation* highlights the new conditions that VCs brought to RD work practices. They described the increased flexibility and a sense of effectiveness with VCs in their work as a “win-win situation” in parallel with the patients benefits. This was interpreted as personal preferences for VC use, motivated by both professional and private considerations. The added flexibility enabled different working locations geographically and allowed them to structure work according to their life circumstances. RDs also reported that VCs had influenced patients’ ability to keep their appointments, adding predictability and improving the effectiveness of their work. RDs expressed the wish that this additional control over their working situation would remain even after pandemic restrictions were lifted.

Some of the RDs described VCs as a way of increasing flexibility in their everyday lives, enabling them to work and live wherever they wanted to, since VCs allowed them to work from home. As with patients, time saved by not commuting was mentioned as an improvement beyond the immediate environmental benefits. Having more flexible working hours as a future possibility when working from home was mentioned by a few RDs, allowing them to work before or after the regular office hours of the healthcare unit to increase flexibility for both RDs and patients. Using the new benefits of increased flexibility and control responsibly was important; RDs emphasized the importance of maintaining professional discipline and doing a serious job when working from home. Concerns had been expressed in 2021 that management would, at some point, withdraw the trust of having colleagues working from home, and that the widespread use of VCs might decrease after pandemic restrictions were lifted. However, in the 2022 interviews, the routines of working from home had become a permanent feature and sometimes there were increased opportunities to work with VCs. Otherwise, there were no observed differences between the two interview occasions regarding increased flexibility by working from home. Extracts from the interviews with Elisabeth and Ruth illustrate the prevailing perspectives and advancement in digital employments:

I think it [working from home] works great. I mean, it’s incredibly luxurious to basically roll out of bed, turn on the computer, and then you’re at work. It’s really fantastic. No commuting time and just… Yeah, just being able to start the day directly without anything. (Elisabeth, interview 2022)

I am now permanently employed based on the fact that it is still a digital… [based at home]… so to speak. Permanently employed, but the digital aspect will be evaluated once a year. If it can continue digitally, so to speak. (Ruth, interview 2022)

The possibility of working from home was also described as increased flexibility to work when being forced to stay at home for various reasons. For example, when on temporary leave of absence from work, such as sick leave with milder symptoms, or being home with sick children. Minimizing time off from work due to the ability to work from home was described as something positive for both RDs and patients. This was highlighted by one interviewee, working as the sole RD in the healthcare unit, because it helped avoid workloads becoming too high when they returned to the workplace. However, it was thought that some patients might limit these new service provision possibilities by not wanting to switch to VCs. Elisabeth voiced the interpersonal dynamic like this:

The limitation [to use VCs] really comes from the patients when you ask them. I might have called and informed them that I need to stay home or that I have a slight cold or something and suggested that we could do it via video tomorrow instead. Then they might have chosen, ‘No, but I’ll wait until you’re back.’ I think the limitation comes from the patients’ side, if I may say so, rather than from my work’s side. (Elisabeth, interview 2021)

By comparison with FTFCs, the increased flexibility of VCs was also seen as contributing to increased effectiveness by reducing the frequency of cancellations and rebookings by patients. A VC could still be conducted even if the patient had a mild infection, as was the case for the dietitians, contributing to higher predictability and control of the working situation. Some noted that VCs could be shorter than FTFCs, although others described a similar time frame for both. Mainly, shorter VC consultations were because they involved less “small talk” than FTFCs. However, one RD reflected that lost information from incidental small talk could be essential to a better understanding of the patient narrative and might need to be actively sought in the consultation. A difference between the 2021 and the 2022 interviews was that, during the 2022 interviews, many described an increase in FTFC bookings. This was attributed to both patient requests and perceived FTFC suitability following the lifting of COVID-19 restrictions. In line with this shift, cancellations and rebookings had also increased again. Alice and Julia illustrate their experience of rebookings from the 2021 and the 2022 interview occasion respectively:

…we get higher attendance now [with fewer FTFCs]. Especially for our annual check-ups after surgery, where there isn’t anything specific to handle. They [patients] are more inclined to come and there are fewer rebookings now, compared to before. (Alice, interview 2021)

I could have one more day a week digitally, and then I wouldn’t have so many cancellations in my schedule due to illness. I’m sure of that. But we’re working on it. (Julia, interview 2022)

### Theme 3: Keeping the care process in mind

The theme *Keeping the care process in mind* highlights the varying degrees of caution the RDs expressed towards VCs. While generally holding a positive attitude towards VCs the RDs also expressed strong perceptions of unsuitability for different steps within the care process, or different patient groups, leading them to limit VC use. Instead, they opted for FTFCs or TCs in certain situations. This was motivated by the aim to minimize the risks of VC-related communication lapses, meeting care process requirements, and ensuring patients had sufficient video literacy. This was interpreted as a personal preference intertwined with a professional normative positioning around treatment quality, whereby decisions not to offer VCs in specific situations were justified through alignment with policy frameworks and proven experience.

The need for assessments and reassessments of body composition and anthropometric measurements, such as body weight, was mentioned as a reason for choosing FTFC over VC. This was due to the risk of making inaccurate assessments based on self-reported measurements. Additionally, the Nutrition Intervention part in the Nutrition Care Process was mentioned by some RDs as easier during FTFCs, such as trying out oral nutritional supplements, or reviewing and explaining information material. This was specifically noted by RDs with no access to a “share screen” function during VCs. The preference for inviting patients to FTFCs for more hands-on steps in the care process remained during the 2022 interviews as well. The possible risks associated with VCs and the perceived benefits of FTFCs were described by Annie and Julia as follows:

It is those occasions when one would need to meet in-person to weigh or measure the patient, as it is difficult to obtain that information otherwise, or one feels that one cannot trust the information received [from the patient], and would actually want to see those numbers with one’s own eyes. (Annie, interview 2021)

If you need to do a proper nutritional assessment, when you need to weigh the patient, maybe measure their height, waist, upper arm circumference, calf circumference, then you often need a physical consultation. Because it can be difficult for patients to use a tape measure themselves, or they might have a broken scale at home. They don’t have a height measuring stick. Then you might need to meet at the clinic. (Julia, interview 2022)

Many RDs were also aware of the potential risk for technical problems or breakdowns with VCs. If audio or video quality deteriorated, or if the connection was lost, they would switch to TC instead. These unplanned transitions were described by some RDs as frequent and time-consuming, detracting from the valuable time allocated for patient interactions. This feeling of insecurity regarding technical functionality remained in the 2022 interviews and was even enhanced for some RDs because their wider VC experience had made them aware of new problems, prompting the use of FTFCs or TCs.

Some RDs experienced an interpersonal disconnect with patients when using VCs, noting that key aspects of communication, such as body language, were more difficult than in FTFCs. This loss of body language was particularly critical when interacting with patients who had cognitive impairments, or limited conversational proficiency because Swedish was not their first language. RDs found it easier to understand and be understood by patients during FTFCs. The sense of disconnect with patients was reduced as RDs and patients gained more experience with VC.

Limited Swedish language proficiency also posed challenges with written information, such as invitations for VCs, which were only available in Swedish. Patients might not realize that the consultation would be held digitally and instead go to the healthcare centre, making FTFCs the preferred choice for sessions requiring language interpreters. Although these sometimes required longer waiting times, some RDs reported using tripartite TCs instead of having one party by VC and the other one by FTFC or TC. In 2021 many of the RDs reported that it was not possible to invite more than one party to a VC. When wishing to invite others to the consultation such as language interpreters, other healthcare staff, or relatives, they had to use FTFCs or TCs instead of VCs. Some healthcare regions had the technical capability to conduct multi-party VCs. However, this was sometimes hindered by safety restrictions or inappropriate booking procedures, necessitating the use of TCs or FTFCs. By the time of the 2022 interviews, more RDs had the opportunity to invite multiple parties to a VC, although some preferred using multi-party TCs instead. The issue of providing information in the appropriate language persisted, prompting the use of FTFCs. The limiting aspects of VCs were outlined by Rita from the 2021 and the 2022 interview occasion respectively:

But there is still a certain distance when you meet on a screen and only see a face and not a whole person. You lose some things, like body language and other aspects, which are also part of communication. (Rita, interview 2021)

If there are language barriers and hearing difficulties, and such, it can also be easier to meet in person. (Rita, interview 2022)

The uncertainty regarding patient VC literacy, meaning the ability to manage technical equipment to connect to VCs, was considered by the RDs as a tipping point for choosing FTFCs or TCs. The patient’s possession of the necessary technical equipment, and user skills, to perform VCs was crucial. The group of patients most frequently mentioned by the RDs as having lower levels of video literacy were older adults. This group was instead invited to meet the RD through TC, either directly or via their contact person. FTFCs were also described as a common alternative for older adults to meet RDs, although these were drastically limited due to the risk of disease transmission during the pandemic. Uncertainty regarding VC literacy among other involved actors, such as language interpreters’ ability to connect to VCs, was also mentioned. Some RDs reflected on how their preconceived notions about patient groups often influenced the choice of consultation modalities due to uncertainty about VC literacy. They emphasized that it was not their intention to differentiate patients based on characteristics such as age, but these were legitimate concerns for service provision. RDs acknowledged that the consultation modality choices for these groups were often made routinely and sometimes needed revision if the patient expressed a preference for VCs instead. This was more frequently mentioned in the 2022 interviews, with some RDs having to revise their preconceived notions of VC appropriateness for certain groups and starting to see it as a question of appropriateness for individuals instead. Some RDs had more actively tried to find out patient VC preference because it could be difficult to predict what an individual patient might be comfortable with. Some RDs called patients before the consultation to check their preference or had an administrator to do this. Reviewing the patient’s history of VC use was also mentioned as a strategy to decide on the consultation modality. The same strategies were mentioned on both interview occasions but, by 2022, clearer routines had been established.

## Discussion

This qualitative study explored how RDs reasoned about the use of VCs following the onset of the COVID-19 pandemic. RDs viewed VCs as contributing to good and accessible healthcare for patients and gaining control over their own working situation, while addressing the importance of keeping the care process in mind. Both internal and external factors — organizational structures, policy frameworks, professional normativity, and individual drivers — were interpreted to shape their views. The RDs in this study all expressed positive views toward VCs, recognizing numerous benefits for patients, professional practice, and their own working conditions. These included increased access to care, fewer rebookings, and reduced travel. Equality in access to care, regardless of location, was frequently mentioned as a key reason for choosing VCs, along with the reduced need for patients to take time off work. However, VCs were considered less suitable for certain care situations and specific patient groups—for example, when anthropometric measurements were required or when consulting with older adults. Additionally, uncertain language proficiency and limitations in technical literacy were reported as reasons for choosing FTFCs or TCs instead of VCs.

Theme 1: *Contributing to Good and Accessible Healthcare* highlights the perceived benefits of VCs for patients, such as saving them time and effort. These findings align with previous research involving other healthcare professionals and their patients [4–8]. One argument in favour of VCs was that they involve less “small talk” than FTFCs, potentially allowing RDs to see more patients in less time. However, as reflected in the interviews, RDs also noted that this could lead to the exclusion of important information and reduce opportunities to build rapport. Showing how consultation modalities may alter the nature of care.

Theme 2: *Gaining Control Over One’s Working Situation* highlights the perceived synergistic benefits of using VCs for patient meetings, including time savings and the flexibility to live in or work from any location. These findings are consistent with prior research showing positive experiences with VCs among healthcare professionals who had adopted the modality [35–37].

Theme 3: *Keeping the Care Process in Mind* reflects RDs’ consideration of the appropriateness of VCs for specific treatment situations and patient groups. Prior studies have shown that VCs are most accepted for follow-up consultations [13, 14]. Research has also pointed to the lack of standardized methods for conducting anthropometric measurements via VCs [39–41].

Efforts to adopt the VC modality in healthcare practice were reflected in the themes of *Contributing to Good and Accessible Healthcare* and *Gaining Control Over One’s Working Situation* by emphasizing the benefits of VCs. These efforts were interpreted as expressions of personal preferences intertwined with normative professional responses aligned with the national healthcare transition towards Good Quality and Local Health Care [24], and global sustainability goals [10]. In these two themes, the increased flexibility and accessibility to healthcare, were promoted by offering VCs as a first-choice consultation modality to patients. The WHO and previous research encourage use of VCs to increase access and decrease inequalities [4–8]. The interviewed RDs spoke of an increase in access to healthcare through VC use. Levesque et al.‘s framework for healthcare access [26], suggests improved *availability* of care to improve the patient’s ability to *reach* care. This was provided by offering VCs, a flexible and geographically independent alternative.

VC effectiveness was argued by reducing traveling and time off from work for patients so limiting the impact on everyday life during dietetic treatment. Benefits for the RDs’ working situation were seen in both clinical and personal terms. These perceived benefits align with VC use perspectives reported by the WHO, among others [4–8, 57]. In relation to Levesque’s conceptual framework perspective [26], RDs perceived increased access to healthcare through the use of VCs, which likely contributed to their greater acceptance of VCs as they recognized and appreciated the benefits. This, in turn, most likely influenced patient’s ability to *seek* care via VCs, further enhancing access to healthcare. Given the risk of infection during the pandemic, the RDs’ *acceptance* of VCs likely increased as they were seen as a safer alternative to FTFCs during pandemic-related restrictions [36, 37, 42].

The perception of *good* healthcare by VCs in theme *Contributing to Good and Accessible Healthcare* was mostly based on the absence of negative feedback from patients regarding VCs. This was interpreted as a way of practicing PCC by accommodating patients’ preferences regarding consultation modality [21]. According to Levesque’s framework model [26], the *appropriateness* of healthcare via VCs, and the ability to *engage* satisfactorily were then decided on non-systematic evaluations and perhaps negatively impacting care outcomes if quality needs were not in fact met. This reasoning for VC use leaving the quality needs of the Nutritional Care Process unattended [29].

Working from home, and thereby more efficiently utilizing working hours, as presented in theme Gaining Control Over One’s Working Situation, may offer benefits but also poses potential risks to the healthcare professional’s work-life boundaries. When both patients and RDs can conduct VCs while experiencing mild infections, and patients were seen to better keep their appointments with VCs, RDs’ experienced better predictability and a feeling of control of the working situation. Pandemic restrictions were, of course, largely the reason that VCs were offered in the first place [35, 41]. In addition, our results indicated more rebookings when FTFCs were available again, after restrictions were lifted. Regardless of reason, VCs added a sense of predictability and effectiveness to the RD work schedule, also seen in prior research [58]. This could be interpreted as contributing to a PCHC approach by including the perspective of the healthcare professionals as well [22]. Again, referring to Levesque’s conceptual framework [26], RD *acceptance* of VCs in this study was most likely enhanced as a result of the personal gain effects of using VCs, such as working from home, and living and working wherever they wanted. This perhaps positively impacted patients’ ability to *seek* VC care. Promoting VCs to patients more often, because of the higher RD *acceptance* in this study, improved *approachability* and ability to *perceive* the option of VC care together with increased access. A few RDs in this study mentioned the potential for even more flexible working hours, with evenings and weekends, in the future because of home working. This was recognised as both a personal opportunity and as increasing flexibility and availability for the patients. However, while the ability to work remotely may offer significant benefits for both healthcare professionals and their patients, earlier research show that it could also blur the boundaries between work and personal life—especially if working hours extend into evenings and weekends. In a study by de Laat, women in particular were showing a tendency to work more when the opportunity was given, further blurring the life-work boundaries [59]. Dietitians in Sweden being mostly women could benefit from being aware of the downsides of the working from home opportunity.

The choice of FTFCs or TCs instead of VCs as described in theme *Keeping the Care Process in Mind*, was argued due to ethical concerns regarding digital literacy and limited language proficiency, as well as care quality concerns related to measurement accuracy and intervention performance. This aligns with previous research on barriers to video consultation implementation [37, 39, 41]. Drawing on Levesque’s conceptual framework perspective [26], *access* to VC care is negatively affected by RD views in this study regarding its *appropriateness* according to requirements of the Nutritional Care Process [29], and with the patients’ ability to properly *engage* with VC care delivery. Affecting the *approachability* of VCs by being promoted less, affecting patients’ ability to *perceive* VC care options available. However, by offering FTFCs or TCs instead, *access* to care deficits were compensated for when these were available. As there is no golden standard for conducting anthropometric assessments by VCs or TCs, it can be anticipated that not all measurements can be performed remotely with the same accuracy as with FTFC [41]. This may potentially jeopardize the Nutritional Care Process and treatment outcomes [29]. Just as not all patients can be expected to have the necessary digital equipment for VCs, not all have access to reliable self-assessment tools, such as calibrated weight scales. This makes the handling of anthropometrical assessments an important issue for RDs to navigate in VC settings.

Prior to a first appointment, RDs mentioned that assessing patients’ preferences for VCs, TCs, or FTFCs was challenging, as little was known about their preferences. The perceived patient preference was sometimes based on inquiries from the patients, interpreted as applying a PCC approach [21], however sometimes it was only based on prior experience without seeking individual patient input. Previous research indicates that patients’ experiences of VCs are connected to their expectations of the consultation with healthcare [60]. From the perspective of Levesque’s conceptual framework [26], *access* to healthcare by VCs is influenced by the RD’s perception of patient preference for consultation modality, perhaps because of assumptions regarding VCs *appropriateness* because of some patients lack ability to properly *engage* with VC care. By promoting or not-promoting VCs, approachability was impacted, influencing patients’ ability to perceive the availability of care by VCs. Seeking to develop VC use while remaining attentive to care quality appears to be a balanced and potentially more sustainable long-term approach.

### Strengths and weaknesses

The study design of Qualitative Longitudinal Research (QLR) was chosen with the intention of observing changes over time, which increases the transferability of the study’s results [45, 46]. However, substantial changes should perhaps not have been expected, as the most considerable change had already occurred with the onset of the pandemic, before the 2021 interviews. Nevertheless, the QLR design contributed rigour through repeated engagement over time, which a single interview occasion could not have provided. The drop-out rate was quite high from the 2021 to the 2022 interview occasion. A certain attrition level was anticipated due to the high workload of RDs. An assessment of systematic attrition indicated that participants who dropped out were similarly positive towards video consultations as those who remained; however, dropouts more often represented infrequent users of VCs. As these interviews were also included in prior analyses, and given our familiarity with the material, we consider it unlikely that this attrition influenced the thematisation of the present paper. The same conclusion goes for the one RD working in private practice. Geographical spread was achieved through the chosen digital recruitment channels, newsletter and social media. Although this study focuses on RDs, the findings may have relevance for other healthcare professions whose care processes are primarily conversational and do not require physical examination, such as psychology or speech therapy [19, 20]. Transferability is, however, contingent upon similar technological prerequisites and organizational conditions, and further research is needed to explore how these insights apply across diverse clinical contexts.

## Conclusion and implications

To conclude, when choosing consultation modality to increase equality, access for remote areas and ad flexibility for healthcare professionals VCs could be a considerable choice of consultation modality. The RDs in this study tended to view VC use in clinical settings according to their perception of patients’ best interests, equality in treatment, and appreciated the personal benefits that followed from home working. This article highlights the value of considering individual circumstances when choosing the most appropriate consultation modality. It also points to the relevance of understanding both patient and healthcare professionals’ preferences, prerequisites and capabilities. Another key consideration is identifying the needs of the care process for the intended consultation and evaluate how different consultation modalities can meet those needs.

When examining changes over time in the results, both consistent patterns and notable shifts were identified. The convenience of not having to travel to see an RD continued to be perceived as a benefit of VCs for patients throughout both interview occasions. RDs consistently reported choosing VCs for patients who demonstrated digital literacy and expressed a preference for this modality. A somewhat stronger positive perception of VCs, and the opportunity to work from home, was reported during the 2022 interviews. However, the need for anthropometric measurements remained a consistent reason for choosing FTFCs at both time points, although such consultations were more limited during the 2021 interview period due to stricter COVID-19 restrictions, which made FTFCs less feasible.

Regardless of who makes the final decision on consultation modality – the patient or the healthcare professional – it appears important that such decisions are informed by consideration of multiple key goals, including personal preferences, person-centredness, sustainability, and the requirements of the care process, particularly when these goals may be in tension with each other. Future research should aim to support healthcare professionals and patients in facilitating this decision-making process. It should also incorporate patient perspectives and explore whether additional individuals, care settings, or treatment needs can be accommodated through VCs, thereby possibly increasing access to care for a wider range of individuals.

## Supplementary Information

Below is the link to the electronic supplementary material.

Supplementary Material 1

Supplementary Material 2

## Data Availability

The datasets used and/or analysed during the current study are available from the corresponding author on reasonable request.

## Abbreviations

FTFC: Face-to-Face Consultation
PCC: Person-Centred Care
PCHC: People-Centred Health Care
PaCC: Patient-Centred Care
RD: Registered Dietitian
TC: Telephone Consultation
VC: Video Consultation
