# Supplementary material for: Dietitian reflections on video consultations: a descriptive qualitative study
Source: BMC Health Serv Res. 2026 May 28;26:768. doi: 10.1186/s12913-026-14818-2 (PMC13217979; doi:10.1186/s12913-026-14818-2)
Supplement: Supplementary file 1 — Supplementary Material 1 [file 12913_2026_14818_MOESM1_ESM.pdf]

Interview Guide 2021

Introduction

- Welcome and brief greeting
- Information about the interviewer
- Brief overview of the study
- Reminder about informed consent, voluntary participation, and the right to withdraw at any time

Background

- Years of clinical experience
- Type of unit
- County
- Patient groups you work with (e.g., diagnoses, children/adults)
- Duration at current workplace
- Do you work alone or in a team?
    - To what extent can you influence your daily work and how patient meetings are conducted?

In-depth – Work and Patient Meetings: Then and Now

- How has the pandemic affected your work and patient interactions?
- What is the most common form of patient meeting today (e.g., face-to-face, digital)?
- Frequency of video consultations
    - Can you describe the process of a digital patient meeting during the pandemic, from referral/contact to completion? (Platform used, identification process, etc.)
    - What tools do you use before, during, and after the meeting? (Analog/digital tools, images, examples)
- Before the pandemic, what was the most common form of patient meeting?
    - Can you describe how you worked previously?
    - What did a typical meeting look like? (Include informal social interactions if relevant)
    - What tools did you use before, during, and after the meeting?

Comparisons

- What advantages/disadvantages do you see in your meeting routines now vs. before?
- What advantages/disadvantages do you see in the tools used now vs. before?
- Have you noticed any changes in patterns or phenomena that are expressed differently?

Time and Logistics

- Time spent per meeting
- Frequency of cancellations/reschedulings (e.g., use of SMS reminders)
- Pre- and post-work
- Overview of patient flow

User Experience – Video Consultations

- In the study invitation, we asked: Do you feel frustrated about the situation? Or are you pleased with the digital progress?
  When registering, you indicated your general attitude toward video consultations as: Positive / Neutral / Negative
  - Is this still how you would describe your attitude today?
  - Can you elaborate? (Assumptions, expectations, experiences)
- Does your attitude vary depending on perspective?
  - From the **patient's perspective** (e.g., equal care, accessibility, treatment quality)
  - From your **professional perspective** as a dietitian (e.g., work environment, professional practice)
  - From your **personal perspective** (e.g., personality, lifestyle, movement)
  - From the **employer's perspective** (e.g., logistics, finances, efficiency, patient flow)
- Follow-up questions:
  - How would you describe presence during video consultations?
    - What do you consider when meeting a patient digitally for the first time?
    - How do you build trust and connection in video consultations?
  - Can you share an example of an unexpected situation during a digital meeting? (Positive/Negative)
    - Interruptions
    - Technical issues
    - Inexperience with technology
    - Work-arounds
  - Have you encountered patients who refuse or are reluctant to use video consultations?
    - How do you handle such situations?
  - Have video consultations affected your work in other ways?
    - If yes, how? (e.g., fatigue, travel, other factors)
  - Have video consultations affected treatment conditions?
    - If yes, how?
    - If no, can you elaborate?

Future

- When the pandemic is over, how would you to work?
    - Similar to before?
    - Similar to during the pandemic?
    - A mix?
    - Other?

Closing

- Is there anything else you'd like to add?

Thank you!
