# Supplementary material for: Dietitian reflections on video consultations: a descriptive qualitative study
Source: BMC Health Serv Res. 2026 May 28;26:768. doi: 10.1186/s12913-026-14818-2 (PMC13217979; doi:10.1186/s12913-026-14818-2)
Supplement: Supplementary file 2 — Supplementary Material 2 [file 12913_2026_14818_MOESM2_ESM.pdf]

Interview Guide 2022:

DESCRIPTION OF THE WORKPLACE

- We last met in spring 2021. Are you still at the same workplace or have you changed jobs?
- Could you describe your current workplace and assignment? Has anything changed since spring 2021?
    - (If new): How long have you worked there?
- How has your work situation changed now that COVID restrictions are being phased out?
    - Physical meetings / Digital meetings / Phone meetings
    - Working from home
    - Commuting
    - Protective equipment
    - Collegial exchange
        - If changed, why do you think that is?

ATTITUDE *(Your attitude in 2021 was positive/neutral/negative)*

Your attitude

- How would you describe your general attitude toward digital meetings today (positive/neutral/negative)?
    - (If different): Why has it changed?
    - What makes you feel that way? (most positive/most negative aspects)
    - Has your attitude strengthened over the past year?
    - How do you compare phone, digital, and physical meetings? When is each used? Are any considered equivalent? (digital/physical or digital/phone)

Colleagues' attitude

- How do you perceive your colleagues' approach to digital meetings? (dietitians/other healthcare staff – embracing, avoiding, organizing/cooperating around them)
    - How do they compare phone, digital, and physical meetings? When is each used? Are any considered equivalent?

Organization's attitude

- How do you perceive the organization's approach to digital meetings?
    - Are patients offered the option of digital meetings?
    - Does the organization support dietitians with equipment, conditions, and adaptations?
    - Have your conditions changed since spring 2021? (equipment, workplace setup, training, etc.)
    - How does the organization compare phone, digital, and physical meetings? What is recommended when? Are any considered equivalent (digital/physical or digital/phone – cost-wise or by directive)?

MOTIVATIONAL INTERVIEWING – Do you use motivational interviewing?

- If yes, how do you use motivational interviewing in digital meetings?
  - Are these conversations different from those held in person or by phone?
  - Do you use any aids? (If yes): Which ones?
  - Do you see any advantages or limitations when it's digital?

ENVIRONMENT

- What kind of environment is the patient in during the conversation?
  - Have you ever felt uncomfortable with the situation, for example if someone else was present or in the background?
- What kind of environment are you in during the conversation?
  - Can the patient tell that you're working from home?

MULTI-PARTY MEETINGS

- Do you conduct digital meetings with multiple parties? (relatives/guardians/other healthcare staff)
- If no, why not?
- If yes, can you describe how you arrange a digital meeting with multiple parties?
  - Any changed conditions compared to last year?
  - How do you conduct multi-party conversations?
  - Is it possible to have multi-party meetings digitally?
  - Is it possible to hold group sessions?
- Based on your experience, how do you perceive the situation of digital multi-party meetings?

FUTURE

- Based on your experiences with digital meetings over the past year(s), how would you like to work moving forward and why? (hybrid, mainly physical meetings/digital meetings)
- Based on your experiences with digital meetings over the past year(s), how do you think patients would prefer to meet with dietitians in the future? (Do you think there are differences between your patient groups? If so, why?)
